# Implementing a Multidisciplinary Tumor Board in the Community Practice Setting

**DOI:** 10.3390/diagnostics7040055

**Published:** 2017-10-17

**Authors:** Michele Lesslie, Jay R. Parikh

**Affiliations:** Department of Radiology, MD Anderson Cancer Center, 1515 Holcombe Blvd—Unit 1350, Houston, TX 77030, USA; JRParikh@mdanderson.org

**Keywords:** tumor board, multidisciplinary, implementation, academics, community

## Abstract

Multidisciplinary tumor boards are an opportunity for radiologists to demonstrate value to referring clinicians, the hospital, and patients. Multidisciplinary tumor boards are commonly utilized in academic institutions, but may not be readily available in community practice. We discuss strategies academic radiologists may employ to assist in the implementation of a multidisciplinary tumor board in the community practice setting. Summary: Strategies to assist in the implementation of a multidisciplinary tumor board in the community practice setting are described.

## 1. Introduction

In response to changes in the health care economic climate over the past decade, academic radiology has been expanding into the community. This community expansion may be achieved through a brick and mortar approach, practice/hospital acquisition, or a partnership-based model in which the community hospital provides use of its facilities, staff, and equipment in exchange for physician staffing by the academic institution [1,2]. As a result, advanced academic radiology services, such as subspecialty expertise and access to cutting-edge technologies, that have traditionally only been accessible at academic medical centers, are made available to the patient population in the community setting [1]. 

A multidisciplinary approach to the care of cancer patients is commonly incorporated in academic institutions and is often achieved through multidisciplinary tumor board (MTB) conferences. MTBs are formal, regularly-scheduled meetings in which networks of specialists devoted to the care of cancer patients meet to review individual cancer patients in a prospective manner to discuss the diagnosis and formulate management plans using an evidence-based approach. These meetings typically involve core groups of medical oncologists, radiation oncologists, surgeons, radiologists, and pathologists, as well as other ancillary members of the healthcare team and other allied healthcare professionals. The patients discussed at these meetings may be patients newly-diagnosed with cancer, or may even involve patients at high risk for cancer or patients with complex management questions [3]. Evidence has shown that MTBs can improve diagnostic accuracy, adherence to clinical practice guidelines, and some clinical outcomes [4].

Radiologists can be the catalyst for implementation of MTBs. Practice building is a time-consuming, difficult, and underappreciated responsibility for radiologists, even in private practice [5]. It is often a foreign concept to academic radiologists or the new trainees. The integration of radiology into the medical, social, and political fabric of a community hospital is critical for radiologists to demonstrate value and not be a disposable commodity [6]. This is the thrust of the Imaging 3.0 cultural transformation being advocated by the American College of Radiology [7]. Multidisciplinary tumor boards (MTBs) represent a tremendous opportunity for radiologists to demonstrate value in the community setting [3]. In community settings where there is no MTB, radiologists can effectively demonstrate their commitment to become p-art of the core culture of a community hospital by initiating the process involved in implementing a MTB. This article is a primer of strategies radiologists can deploy to assist in the implementation of a MTB in the community setting. Specifically, we focus on practical suggestions for implementation of MTBs and the value that MTBs bring to the community practice setting.

## 2. Establish the Physician Team 

An enthusiastic radiology leader can actively recruit other interested physicians to build an MTB team. This can be achieved by networking and socializing amongst the members of the community health care team [8,9]. Oftentimes, the idea may be proposed while discussing radiologic findings on a newly diagnosed cancer patient at the Picture Archiving and Communication System (PACS) workstation. The opportunity may arise while determining cancer staging with a medical or radiation oncologist, discussing biopsy results with a pathologist or assisting a surgeon with surgical planning of complex cases that may benefit from a multidisciplinary approach. Interactions could also occur at other interdisciplinary hospital committees such as a credentialing committee or quality assurance committee. 

An important step is determining the physician who will assume the team leadership role as the MTB Chair. This is often an established physician leader in the community hospital and can be from any specialty, including radiology. Designated responsibilities of the Chair include selection of appropriate cases to be discussed at the meeting, presenting the cases, ensuring that the meeting begins and ends in the allotted time and includes relevant and lively discussion. As the team leader, the Chair should keep the discussion on track and encourage active participation by all of the various MTB members [10]. Patient confidentiality must be maintained by reminding the participants and attendees at each meeting.

Additionally, it is important to assemble a willing team of health care professionals that will commit to attending regular MTB meetings and will also refer cancer patients that could benefit from discussion in the MTB. As with all teams, the MTB team should consist of the right mix and number of members [9]. Evidence has shown that multidisciplinary teams can impart improvements in clinical care by consensual decision-making and effective teamwork [11]. Suggested team members (if available) include diagnostic radiologists, pathologists, surgeons, medical oncologists, radiation oncologists, nuclear medicine, a cancer program administrator or cancer registrar, oncology nurses, social work, psychiatrists, palliative care, nutritional services, physical/occupational therapy, and primary care physicians. These interdisciplinary teams offer the opportunity of multiple expert opinions working in a coordinated, patient-centric fashion [12]. 

## 3. Administrative Assistance

It is particularly beneficial to have the support of a MTB coordinator, such as the cancer registry manager, who can be responsible for the administrative management and organization of the logistics of the meeting. Newly diagnosed or recurrent cancer cases and the proposed treatment plan should be forwarded to the MTB coordinator. The MCC chair or lead physician should decide which cases will be presented, as not every case forwarded warrants a detailed discussion. Once the patients to be presented are selected, the MTB coordinator should create a list of patient cases to be presented and invite the clinicians directly involved in the care of each patient being presented. A meeting room should be booked. Breakfast or lunch may be organized. Marketing with emails to health care professionals in the community and fliers placed in the hospital may also augment attendance. A designated coordinator should assist with data requirements, such as recording attendance, tracking the number of cases discussed by disease site and the MTB treatment recommendations. It is also important to organize continued medical education (CME) to attendees.

## 4. Logistics

Institutional support is critical to the implementation and maintenance of tumor board meetings. First and foremost, a suitable meeting room with appropriate facilities must be available. It is preferential to have weekly or monthly meetings on a set, agreed-upon day and time that can accommodate most professionals’ busy schedules and ensure that the key physician champions and administrative support teammates can be present to facilitate discussion.

The availability of proper imaging, histopathological information, and clinical information is imperative for effective decision making [13]. The ability to connect to the patient database for the hospital and/or network is useful to display and discuss this information. Projection equipment is needed to display imaging and pathology slides. A concise presentation, which may in Power Point format, may be used to organize images and facilitate discussion. In community settings that may not include a full range of specialists in-house, teleconferencing or videoconferencing could be an option to ensure that the expert opinions of all necessary specialists is available. Given all the necessary audio/visual and computer equipment and the potential for malfunction, information technology (IT) support is indispensable. It is crucial that patient confidentiality be maintained throughout the discussion, including anonymizing radiologic and pathology images, as well as the clinical documents that are presented.

The majority of cases are usually newly-diagnosed cancer patients, but may also involve cases of recurrent cancer, high risk benign cases, such as dysplasia or atypia, or previously reviewed cases that may require additional follow-up [14]. 

## 5. Marketing

Studies have shown that there is variability in perceived benefits amongst providers regarding MTBs [15]. During the implementation process, radiologists have the opportunity to discuss and market the MTBs with referring clinicians, such as family physicians, internists, and obstetrician/gynecologists. This can be in the physician lounge, hospital functions, and community events. Marketing with emails to health care professionals in the community and fliers placed in the hospital may also augment attendance. Ultimately, formalized institutional polices and tumor board agendas could be designed to engage chronic low-frequency attendees [16]. 

## 6. Social Value of Tumor Board

Beyond the clinical benefits that can be gained from MTBs, there is also a social value to participating with members of the community health care team in a multidisciplinary setting. MTBs provide an ideal opportunity for academic radiologists to interface directly with referring clinicians and show a willingness to become an engaged member of the professional community and to collaborate with healthcare providers in other disciplines. Academic radiologists earn the confidence of referring physicians and increase their personal job satisfaction through direct interaction with the multidisciplinary team. Building trust and fostering relationships with clinicians and other healthcare professionals at the community level are essential steps in demonstrating the relevance of academic radiologists to the professional community [3]. Through MTB leadership or active participation, radiologists can gain credibility with hospital administrators. 

Breakfast or lunch may be organized with the MTBs. This not only serves as a marketing tool, but potentially facilitates social interaction at the conference. The Chair should be cognizant of the social value of the conference for all participants, and allow for some informal dialogue during the conference to give it a more personal touch. Networking and socializing with other professionals can be an important resource for the exchange of information and may also help create loyalty [8].

## 7. Education

MTB attendance can provide valuable CME credits for physicians, as well as continuing education credit for nurses, technologists, and practitioners in other disciplines. Regular participation in departmental or group conferences (at least 10 per year) focused on patient safety, including attendance at MTBs meets the requirements of Part 4 of the updated American Board of Radiology Maintenance of Certifications (Practice Quality Improvement) [17]. MTBs are valuable avenues for education for health care teams. Currently, none of the Accreditation Council for Graduate Medical Education (ACGME) requirements for any oncology program mandate spending time in other oncology fields [18]. Therefore, as opposed to blaming those who lack multidisciplinary knowledge for their deficits, the root cause may lie in their training. In order to use an evidence-based approach to make informed decisions regarding cancer patient management, members of the health care team involved in the MTB must stay updated on current evidence-based guidelines and remain apprised of the recent medical literature. MTBs are forums for the discussion and implementation of clinical practice guidelines, such as those of the National Comprehensive Cancer Network (NCCN) [19].

## 8. Quality and Safety

The enhanced level of communication among members of the health care team during an MTB should ideally lead to improved patient quality and safety. For decades, tumor boards have been an established part of the care of cancer patients [20]. Differences in radiologic interpretation, pathologic interpretation and clinical evaluation by surgical, medical, and radiation oncologists can be influenced by the team approach afforded by MTBs [21]. MTBs have been shown to impact management decisions of cancer patients. A recent systematic review of the literature (which included 27 articles) demonstrated that between 4% and 45% of patients discussed at MTBs experienced changes in diagnostic reports. Additionally, patients discussed at MTBs were more likely to receive more accurate pre-operative staging and neoadjuvant/adjuvant therapy [22]. The review showed limited evidence of survival outcomes, in contrast to earlier large cohort studies [23]. During the implementation of an MTB, a log should be promptly initiated of changes in treatment. Presenting follow-up data documenting the percentage of changes in patient management from MTB can represent an early win for the program, hospital, and patients. Auditing outcomes of MTBs essential to ensuring the quality of the clinical decision-making and overall function of the MTB. Other countries have also successfully implemented MTBs and incorporated audits. For example, in the United Kingdom, the National Health Service has prioritized audits of clinical decisions from multidisciplinary team (MDT) meetings, and in particular, patients who die within 30 days of MDT discussion [24] Although clinical outcomes are a useful way to measure the effectiveness of MDT performance, other elements may also be used to assess performance, such as the ability to reach a decision, whether or not decisions are implemented, time from diagnosis to treatment, and cost reduction. Additionally, patient satisfaction is increasingly viewed as an important measure of healthcare performance [25].

The American College of Surgeon’s Commission on Cancer Program accreditation requires cancer programs to have a multidisciplinary cancer conference that prospectively reviews cases and discusses management decisions [26]. Hospital leadership therefore has a vested interest in the success of MTBs. Therefore, by championing and implementing MTBs in the community setting, radiologists demonstrate their commitment to quality to referring clinicians, members of the multidisciplinary team, administration, and patients.

## 9. Conclusions

Due to changes in the delivery of health care over the past decade, academic radiology has become, and continues to become, more and more prevalent in the surrounding community. This expansion has resulted in the availability of academic services that are typically present at large academic centers, such as a multidisciplinary approach to cancer care. Multidisciplinary tumor board meetings are a vital component to this approach and have been shown to be influence changes in patient management and treatment plans. Through MTBs, academic radiologists can also collaborate with other physicians and assume a more active role in the community setting. The principles and ideas presented in this article can serve as a guide to initiating a MTB conference in the community, thereby delivering the benefit of an academic multidisciplinary approach to cancer care that may not previously be present in the community practice setting.
